# Meat and Fish Freshness Inspection System Based on Odor Sensing

**DOI:** 10.3390/s121115542

**Published:** 2012-11-09

**Authors:** Najam ul Hasan, Naveed Ejaz, Waleed Ejaz, Hyung Seok Kim

**Affiliations:** 1 Department of Information and Communication Engineering, Sejong University, 98 Gunja-dong, Gwangjin-gu, Seoul 143747, Korea; E-Mails: hasan@sju.ac.kr (N.H.); waleedejaz@sju.ac.kr (W.E.); 2 Department of Digital Content, Sejong University, 98 Gunja-dong, Gwangjin-gu, Seoul 143747, Korea; E-Mail: naveed@sju.ac.kr

**Keywords:** electronic nose, machine olfaction, pattern classification

## Abstract

We propose a method for building a simple electronic nose based on commercially available sensors used to sniff in the market and identify spoiled/contaminated meat stocked for sale in butcher shops. Using a metal oxide semiconductor-based electronic nose, we measured the smell signature from two of the most common meat foods (beef and fish) stored at room temperature. Food samples were divided into two groups: fresh beef with decayed fish and fresh fish with decayed beef. The prime objective was to identify the decayed item using the developed electronic nose. Additionally, we tested the electronic nose using three pattern classification algorithms (artificial neural network, support vector machine and k-nearest neighbor), and compared them based on accuracy, sensitivity, and specificity. The results demonstrate that the k-nearest neighbor algorithm has the highest accuracy.

## 1. Introduction

Meat and its products are highly subject to spoilage and contamination due to bacterial effects [1]. Eating such spoiled or contaminated meat can cause severe health hazards. To avoid such a condition and to safeguard public heath, every government should make it a priority to develop techniques to guarantee the safety/quality of meat. To cope with this problem, there are laws in almost every part of the World that prohibit the sale of diseased/spoiled meat. It is also assumed that butchers are always competent enough to make this determination. To enforce the implementation of this system, government agencies usually appoint food inspection officers to visit butcher shops to gauge the quality of the meat. The inspection officer's duty is to report when a butcher is selling such spoiled meat. Based on the report generated by the officer, the veterinary advisor takes legal action against the dishonest butcher [2]. The primary advantage of this lies in the fact that it tends to make the butcher more careful in the future.

While multidimensional efforts can be made in this direction, the current state-of-the-art method for inspecting butcher shops to ensure the quality of their meat is based on visual appearance and classical olfaction. Classical olfaction technologies including olfactometry and scentometer use trained odor panelists to smell air samples to determine the strength of an odor [3]. Both olfactometry and scentometer methods are expensive to use and are susceptible to the assessor's personal bias [4]. Therefore, unbiased automatic odor evaluating technologies are desirable. Recently, creditworthy efforts have been underway to develop more sophisticated tools for ensuring meat quality by the joint work of the sensor development technology and pattern recognition communities. Through advances in both areas, a tool named an electronic nose has been invented based on the human olfaction method.

An electronic nose is a system that can detect and recognize items on the basis of their odor signature. The system consists of an array of electrochemical sensors and an appropriate pattern classification algorithm to recognize specific odors. A comprehensive review on the history of the electronic nose is provided in [5]. An electrochemical sensor is a device capable of converting a chemical quantity into an electrical signal [6]. Over the last few decades, the advancements and growth in sensor technologies have shown promise for the development of a faster, real-time electronic nose system to ensure meat quality/safety. A review of the current applications and technological advancements of the electronic nose is presented by Wilson and Baietto [7].

Nowadays, the electronic nose is commonly used in numerous fields including the food, environmental, medical, and narcotics industries [8]. In the food industry, the electronic nose has proven to be very effective for a number of purposes such as quality control, process monitoring, freshness evaluation, shelf life investigation, and authenticity assessment. A considerable amount of work has been done to ensure the quality of meat, yet the application of an electronic nose for the inspection of butcher shops remains unexplored. A detailed survey of meat quality assessment using an electronic nose in different environments is discussed in [9]. In the present study, we propose a mechanism to arm the inspection team with an electronic nose to assist them while inspecting butcher shops. The results suggest that an electronic nose is effective at identifying spoiled meat items in butcher shops. The major contributions of this paper are summarized as follows:

- Design of a low-cost and portable electronic nose system that can be employed for food market inspections to identify the spoiled meat out of a number of different types of stocked meat.
- Arm the developed system with different classification algorithm namely artificial neural network (ANN), support vector machine (SVM) and k- nearest neighbor (KNN).
- It describes in detail the development of the system and presents the results of classification algorithms to evaluate the reliability of our E-nose prototype to produce correct measurements.

The remainder of the paper is organized as follows: related background work on artificial olfaction using an electronic nose is reviewed in Section 2. The proposed system for food inspection is described in Section 3. Experimental results to illustrate the performance of the applied pattern recognition techniques are shown in Section 4. Finally, concluding remarks are given in Section 5.

## 2. Machine Olfaction Using an Electronic Nose

To inspect the quality of food in supermarkets, it is needed to develop rapid methods to identify the spoiled food items such as meat and fish. Nowadays, evaluation of the degree of spoilage of meat is usually subjective, using either sensory assessment or microbiological analysis [9]. Sensory analysis employs human senses to provide information about color, taste, and smell as well as overall degree of spoilage. However, major drawbacks of this method are high cost of training the human panel, lack of high degree of reproducibility of evaluation, and standardization of the measurement. The most common biological method to determine the meat spoilage is a bacteriological method based on a bacterial count. However, there is a lack of the correlation between the degree of spoilage and the bacterial counts, which is often observed [10]. Furthermore, microbiological analyses are time consuming, destructive to tested products, and do not provide ‘the immediate answer required’ to the industry [11]. It is thus crucial to replace these with a method which is faster and applicable to the real-time identification of spoiled meat.

Meat spoilage/contamination is obvious when off-odors, off-flavors, or discoloration are present. Therefore, odor/smell signatures are considered a good source of information for determining spoiled items. The main sensory system used by humans to sense the odor of a particular substance is olfaction; the organs involved in olfaction are the nose and the brain. This has led researchers to attempt to mimic the human nose system to identify spoiled items based on their odor. As a result, the electronic nose—an instrument performing odor analysis—derives its name from the human nose, and the process is known as machine olfaction. A detailed analogy between human and machine olfaction is discussed by Ghasemi *et al.* [12].

Significant progress has also been made to mimic other human sensory organs such as the eyes, ears, and skin. However, efforts to mimic the human nose are still inadequate and in the preliminary stage [13]. Moreover, most of the commercially available instruments mimicking the human nose are very expensive and difficult to maintain [14]. This study focuses on building a simple, low-cost electronic nose to engage in real-time sniffing of the meat market and to identify spoiled meat.

Research over the last few decades has revealed that machine olfaction is the most accurate and consistent means of odor analysis. Human olfaction is based on the chemical interaction of volatile odor compounds and neurons in the nasal cavity. The signals generated by the neurons are transferred to the brain for further recognition of a particular substance. In machine olfaction, the sensors are equivalent to human neurons and the pattern-matching algorithm is similar to the brain's recognition process.

Noteworthy efforts have been made in recent years to improve machine olfaction sensor technology as well as pattern-matching algorithms. With the advances in sensor technology, several types of chemosensors are currently available, but the most common are the metal oxide semiconductor (MOS), metal oxide semiconductor field effect transistor (MOSFET), conducting organic polymer (CP), and piezoelectric crystals [7]. A comprehensive survey on the advancement of machine olfaction sensor technology is presented in [15]. In this study, our focus is on the electronic nose developed for meat spoilage analysis using metal oxide semiconductor technology.

In [16], the authors proposed a MOS-based electronic nose to measure the volatile signatures associated with food quality, and then discriminated meat into spoiled and unspoiled categories. This scheme uses a support vector machine as the pattern classification algorithm. Similar work was conducted in [17] in which the authors proposed a neural network-based metal oxide artificial olfaction method to differentiate between spoiled and unspoiled food. However, the primary concern was quality control in food production facilities rather than identifying off-odor substances.

In [18], the authors proposed a mechanism for monitoring the spoilage of meat inside a refrigerator. This particular electronic nose uses a sensor array of 10 MOSFET and four MOS sensors. This scheme predicts, in days, the time within which a sample of meat that has been stored in the refrigerator will spoil. It uses beef and pork samples for the experiment and an artificial neural network as a prediction algorithm. In [19], the authors have shown the feasibility of using electronic noses in the poultry industry. Similarly, in [20], the authors experimented with electronic noses in dairy products stocked in a smart home, such as milk and yogurt.

A substantial work is also reported in the literature regarding the freshness of fish using electronic nose. For instance, the authors in [21] presented a portable electronic nose made up of tinoxide sensors to classify the fish into spoiled and unspoiled categories both in refrigerator as well as room temperature. Similarly, the authors in [22] proposed an automatic method to inspect the quality of tuna fish. However, this inspection method is based on visual color information and the scheme only analyzes the quality of tuna fish at the production level.

As described, considerable work on individual beef and fish spoilage identification has already been conducted. Therefore, additional research is necessary to develop an electronic nose that considers multiple items simultaneously. The objective of this study is to develop an instrument for machine olfaction that can act as an assistive tool for food inspection teams, thus increasing the accuracy and efficiency of the inspection process. Instead of categorizing a single substance into spoiled and unspoiled groups, this instrument can detect spoiled meat from a number of different types of meats. In addition, a comparison was conducted using different pattern classification algorithms to determine the instrument's applicability to the practical environment.

## 3. Description of the System

**Figure 1.**
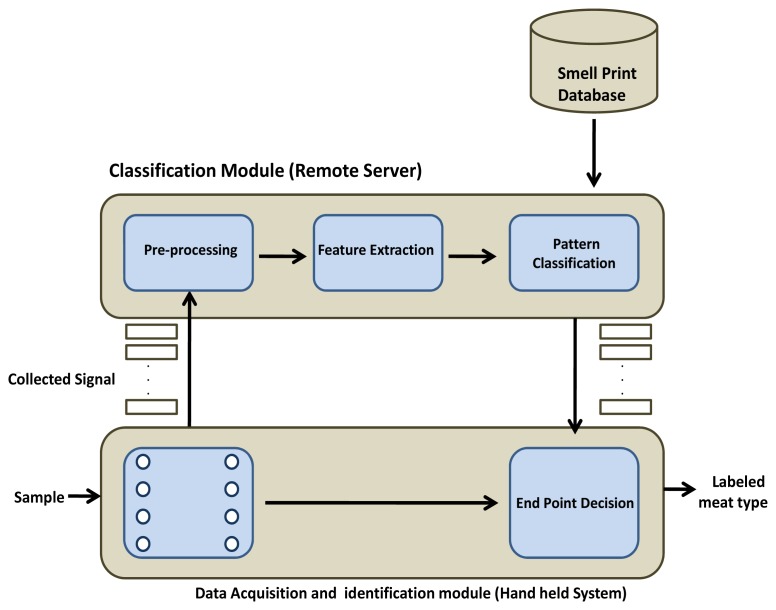
The overall block diagram of the electronic nose for the identification of spoiled meat.

In this paper, we focus on devices that can be used to sniff in the retail market, based on sensor technology as well as machine learning solutions. The electronic nose, held in the hand of the food-inspection officer, smells the shop and identifies spoiled items. More specifically in our case, a butcher shop is examined. As depicted in Figure 1, the proposed system consists of two major modules: classification and identification.

The data acquisition and identification module is performed in the hand-held device that is carried by the inspecting officer, whereas the classification module is done with a remote server. After acquisition, data are transmitted to the remote server for further processing through a Wi-Fi or cellular communication module that is mounted on the top of the hand-held device. Moreover, a display is also equipped and shows the sensed signal that is being transmitted to the remote server. Further details of both modules are mentioned below.

### 3.1. Data Acquisition and Identification Module

**Figure 2.**
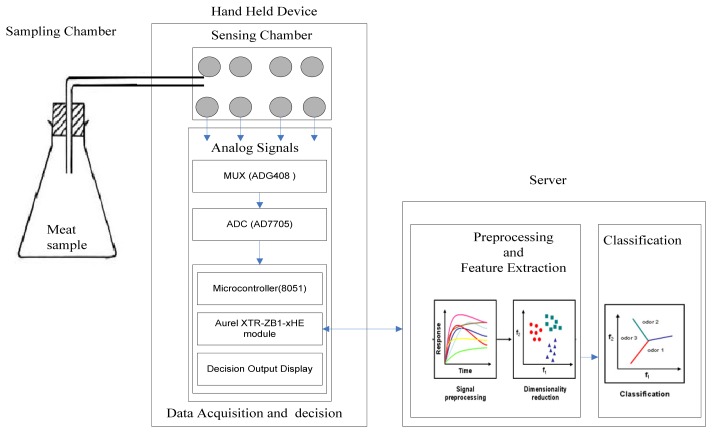
Electronic nose system.

The developed hand-held system is responsible for data acquisition and identification of the type of spoiled meat. It is made up of three components, namely sensor array, communication equipment, and the end point decision. Figure 2 shows a diagram of the developed electronic nose system.

A conical flask of 600 mL serves as a sampling chamber. The conical flask is closed with a rubber stopper. The rubber stopper consists of a hole with a pipe. The pipe goes to the sensing chamber. The sensing chamber has 8.5 cm × 12 cm × 3 cm dimensions, is airtight and was constructed from Perspex^®^ glass, which is non-reactive to chemical or food vapors. The odor samples from the studied meat samples are collected from the sampling chamber, and then, injected into the sensing chamber through an autosampler at 120 mL/s. Therefore, the turnover time (T) for one sample can be calculated as the ratio of the capacity C of a sampling chamber (conical flask), *i.e.*, C = 600 mL and the total rate of removal S from the flask (*i.e.*, S = 120 mL/s): T = C/ S = 600 mL/120 mL/s = 5 s. The sensing chamber consists of a sensor array, temperature control circuit. The temperature control circuit provides feedback voltage to the heater of sensors so as to guarantee that the sensors are at stable temperature. In this study, the sensor array in the developed system consists of eight semiconductor gas sensors. When compared to other electrochemical gas sensors, semiconductor gas sensors offer several advantages, including the simple principle of operation, low manufacturing costs, commercial availability, and small size.

A gas sensor array is used when the smell is composed of a mixture of various gases. Indeed, in this case, a gas sensor array can perform better than a single sensor even for the concentration measurement of a single gas component. The sensor array comprises eight MOS sensors. Five sensors including GSLS61, GSAP61, GSBT11, GSET11, GSNT11 from Ogam Technology and the remaining three sensors MQ3, MiCS-2610, TGS 826 are from Futurlec, e2v and Figaro Engineering, respectively. Eight types of quantities are measured using these sensors, including oxidizing gas O_3_, liquid petroleum gas/natural gas (LPG/NG), nitrogen oxide (NOx), alcohol, smoke, and volatile organic compounds (VOC) such as carbon dioxide (CO_2_), carbon monoxide (CO) and ammonia (NH_3_) gas. Table 1 provides a list of measurable quantities as well as the identification codes of the sensors used in the developed system to measure these quantities.

The sensor response signal is fed to the 8051 microcontroller via a multiplexer MUX (ADG408) for the serial interface and 16-bit analog to digital converter ADC (AD7705). The microcontroller is mounted with a ZigBee module (Aurel XTR-ZBI-xHE) and a display. Each sensor output is transmitted to a remote server through the ZigBee module embedded in the microcontroller. After processing the received data, the remote server sends back the classification result to the hand-held device. Finally, the hand-held device produces the label for the meat samples based on the classification result.

### 3.2. Classification Module

The classification module plays the most decisive role in obtaining a versatile instrument able to reliably recognize a wide variety of odors [23]. The classification process can be divided into three steps: preprocessing, feature extraction, and pattern classification.

#### 3.2.1. Preprocessing and Feature Extraction

The preprocessing step involves noise elimination and filters the data received from the developed electronic nose. The signal *versus* time response for all the sensors for both decayed beef and fish has presented in our previous work in [24]. For the sake of simplicity and low computational cost, a moving average finite impulse response is used, mathematically, it can be written as:

(1) $$x[n]=\sum_{i=0}^{M}b_{i}x[n-i]$$

(2) $$b_{i}=\frac{1}{M+1}$$

where x[n] is the sensor response that needs to be denoised; M is the order of the filter. The higher the order of the filter the smoother the curve will be, but it will also lead to a loss of details. In this study we chose M = 3. In feature extraction the main task is to reduce the number of dimensions to extract the most critical information. There are two kinds of features which can be extracted from the denoised sensor response curve: transient state and steady state. Although the whole denoised sensor curve can be used in feature extraction, the steady state response is more often used due to its robustness. In this study, we are also using the steady state response of the denoised sensor response curve for the feature extraction. The maximum absolute value of each denoised sensor response during the steady state is obtained, therefore, after the feature extraction process, a row vector with eight elements is obtained, corresponding to each sensor output. Finally, the last step is pattern classification, which produces information based on the matching of the row vector with the smell prints of different kinds of meat stored in the database. The different classifiers employed for matching and how they work is discussed in the subsequent section.

#### 3.2.2. Classifiers

We examined three statistical learning algorithms, namely artificial neural networks (ANN), support vector machines (SVM), and K-nearest neighbor (KNN), which are used to identify spoiled food items. Training and evaluation for classification use two main classes, namely beef and fish.

##### 3.2.2.1. Artificial Neural Network (ANN)

An ANN is a learning algorithm that resembles the human brain process [25]. An ANN consists of a large number of processing elements also known as neurons. A transfer function is associated with each neuron, which is used to map the inputs to the outputs of that neuron. Moreover, the neurons are tied to one another using weighted connections. Learning occurs by a set of examples known as training data, *i.e.*, the data for which the ground truth is already known. Based on the training data, the weight assigned to the connection between the neurons is adjusted; this is necessary to solve specific problems [26].

In this paper, we have adopted a multi-layer ANN to identify spoiled meat using an electronic nose. Most researchers have used a three-layered ANN topology since the authors in [27] indicated that it has a sufficient computational degree of freedom to solve classification problems. In the three-layered ANN, the neurons are organized into three distinct groups of elements: input, hidden, and output layers. The number of neurons in the input layer corresponds to sensors in the array, and there are eight sensors in the case of our developed electronic nose. The number of neurons in the hidden layer is determined experimentally, and the number of neurons in the output layer corresponds to the number of classes, which is two in our case, *i.e.*, either rotten beef or fish.

**Figure 3.**
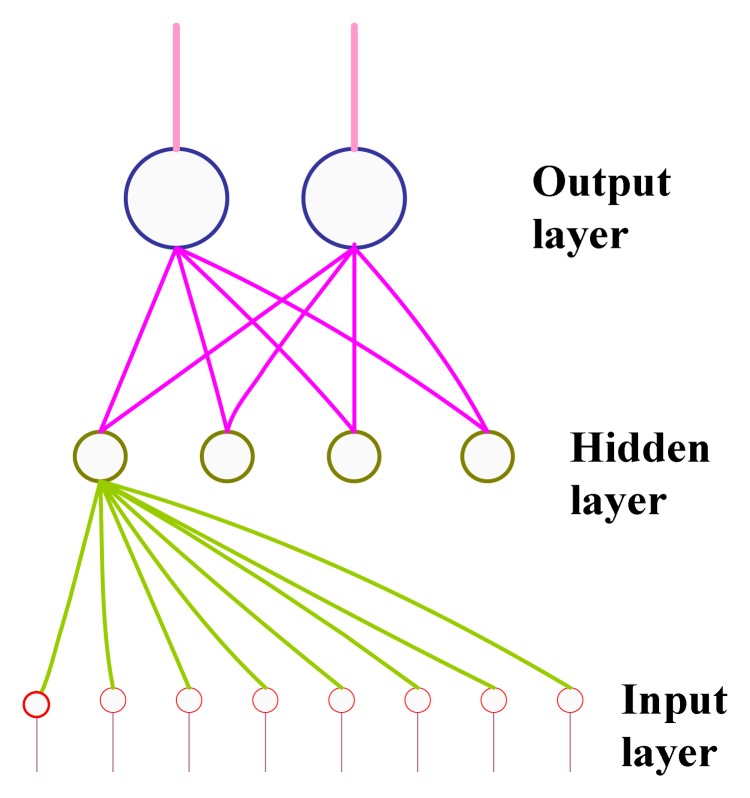
Structure of a fully connected three-layer backpropagation network used to process data from eight MOS sensors into the electronic nose to identify the spoiled meat type, *i.e.*, beef or fish.

One of the most popular ANNs used for odor classification in an electronic nose is a three-layered ANN feedforward backpropagation trained ANN [28,29]. To train the network, it is necessary to provide it with a number of input examples with corresponding output classes (supervised learning). Each neuron adds its weighted inputs and performs a nonlinear transformation, *i.e.*, sigmoid function of this sum. The calculation is carried out for each layer, feeding the values through to the output layer. During the learning phase, the weights are adjusted to minimize the difference between the actual and ideal output. Once the network is trained, it can be used to predict the class label for the test examples. Figure 3 shows the topology of the ANN used to identify the spoiled meat type.

##### 3.2.2.2. Support Vector Machine (SVM)

**Figure 4.**
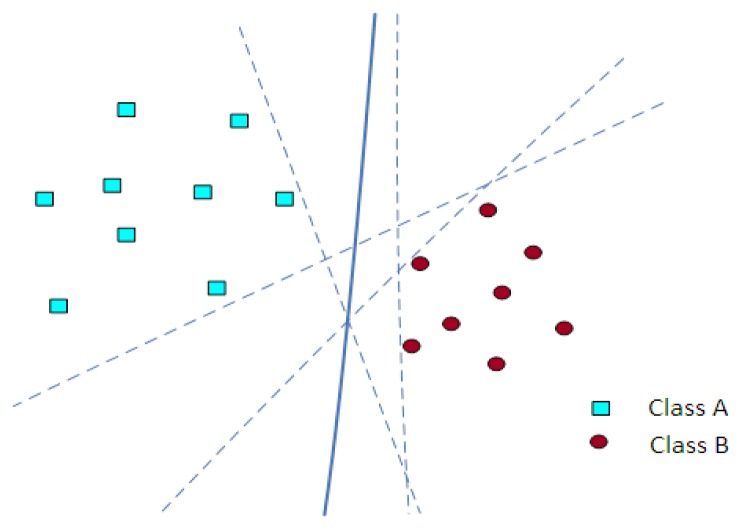
Optimal separating hyperplane.

The support vector machine (SVM) is a well known classification approach in the field of machine learning. SVMs hinge on two main mathematical operations: first, mapping the input data into a high dimensional space using a kernel function, and then separating them into different classes using a hyperplane decision surface [30]. Consider two classes, A and B. Members of class A are represented by squares and members of class B with circles, as shown in Figure 4; the goal is to develop a function deduced from the available examples which can separate the A and B classes. Furthermore, this designed function acts as a classifier for unseen or test examples of either class A or B. There are many possible linear classifiers that can separate the examples of class A and B, but there is only one which maximizes the margin (maximizes the distance between itself and the nearest point of both classes A and B). This classifier which separates the two classes A and B is known as the optimal separating hyperplane. In Figure 4, the optimal separating hyperplane is shown by a solid line.

If that data is not linearly separable, a mapping function known as a kernel maps the input data to a higher space to ease the separation between different classes. SVMs were originally designed for two-class classification. Extending them to work for multiclass classification is still an ongoing research issue [16]. Several techniques have been proposed to build a multiclass classifier using a binary SVM classifier. In [31], the authors suggested that the one-against-one approach to construct a multiclass classifier is the most suitable for practical use. In this strategy, *m*(*m*-1)/2 binary classifiers are built, where *m* is the number of classes and each classifier is trained on data from two classes. There are several ways to combine the output of these classifiers; among them, the most common is the voting mechanism. In this mechanism, every classifier assigns the vote to one of the two classes used in the training, and finally a decision is taken in favor of a certain class that receives the largest number of votes [32].

##### 3.2.2.3. K-Nearest Neighbor (KNN)

K-nearest neighbor (KNN) is a supervised learning classification algorithm. The classification rules are generated based on the training examples without any additional parameters. To classify a test sample, the *K* nearest neighbors in the training data are found using Euclidean distance and labels the test sample with a class name by applying a majority rule among *K* neighbors [33]. Accordingly, the rule applied in this study is: assume that there are m classes with a d-dimensional feature vector X = (x0, x1, …, xd-1) associated with each class and there are N training examples. Each jth training example can be written as Tj (xj0, xj1,…, xjd-1) →Y where Y is the class label. For simplicity, each parameter is set as m = 2, d = 2, and N = 4, which means that there are two classes denoted by A and B, and each class has just two attributes and four training examples in the database. The training examples are set to T1(7,7)→B, T2(7,4)→B, T3(3,4)→A and T4(1,4)→A. Suppose that a query sample Q (uo, u1) = Q (3, 7) is given; the objective is to find the class of the query sample using the KNN algorithm with K = 3. The first step is to compute the distance between the query instance and each of the training samples. The distance between the query and the ith training sample is denoted by Di and mathematically computed as:

(3) $$D_{i}=\sqrt{{\left(x_{io}-u_{o}\right)}^{2}+{\left(x_{i1}-u_{1}\right)}^{2}}$$

The distance of the query sample from each training example obtained are D_1_ = 4, D_2_ = 5, D_3_ = 9, and D_4_ = 2. Because K is 3, three training examples T1, T2, and T4 are chosen as those having the minimum distances from the query instance. Out of three chosen examples, two belong to class B; so the decision is that the query example belongs to class B. The class of the test sample can be determined through KNN mathematically as:

(4) $$c=\underset{i}{\arg\;\max}{\left\{\frac{K_{i}}{K}\right\}}_{i=1,\ldots ,m}$$

where c is the label of the class assigned to the test sample, Ki is the number of nearest neighbors belonging to class I, and K is the total number of nearest neighbors. The training vectors are classified in advance into m classes.

## 4. Experimental Results and Discussion

In this section, the sample preparation and the sampling method used to evaluate the proposed food inspection system are described. Next, implementation details and the algorithmic parameters used in the simulation are defined. In addition, the metrics on which performance is measured are described. Finally, a comparison is conducted among three classifiers: ANN, SVM, and KNN.

### 4.1. Sample Preparation and Sampling

For this study, two kinds of meat samples (beef and fish) were used. Meats decay rapidly compared to other items, and intake of such contaminated foods may cause food poisoning. If decayed meat is exposed to the proposed system by placing it in the sampling chamber, it can detect the combination of the gases generated from the decayed food and identify the spoiled item using a pattern classification algorithm and the database containing the smellprint of different food items. The experiment was conducted at room temperature for seven days. The data were measured after a regular sampling interval of 15 minutes. The cleaning and sampling time of the measurement process were determined based on the obtained sensor responses that have been tested. It was found that 200 s cleaning time and 200 s sampling time were sufficient to clean and to sample the beverages' odor. Thus, the total time required for the cleaning and sampling process are 10 min (600 s = 200 s + 200 s + 200 s). The occurrence of drift in the sensor signals was not measured explicitly, since no measurements with a calibrated gas were performed during the measurement phase. However, no significant response drift was observed during experiments for the raw value of the sensors. Even though some slight fluctuation in the baseline of the sensor raw value could be detected, but identifying a trend was not possible, therefore, drift is neglected in this study. The measured data was then stored in the database at a remote server. To remove noise, data measured by the sensors was preprocessed using a well known moving average filtering technique. After filtering the data, the maximum absolute value measured by each sensor was recorded. As mentioned earlier, our system has eight sensors, therefore; after every sampling interval, there was a row feature vector with eight elements. The collected dataset consists of a total of 1,372 samples of which 784 samples were rotten beef and fresh fish, and the other samples were fresh beef and rotten fish. We carried out the experiment such that either beef or fish was rotten. Furthermore, we divided the dataset into two parts: training data and testing data. The training data includes 175 samples of which 100 samples were rotten beef and 75 were rotten fish. The testing data includes 1,197 samples in total, out of which 684 samples were rotten beef and the other 513 samples were rotten fish. The dataset used for the experiment is summarized in Table 2.

### 4.2. Parameter Setting

First, a multi-layer perceptron feed-forward ANN classifier was used to test the ability of the electronic nose setup to identify the spoiled meat. The feed-forward neural network architecture consists of input, output, and one or more hidden layers. Each layer has a predefined number of neurons. In this study, we defined the neural-network architecture with only one hidden layer. The input layer serves as a buffer for the input; the input layer has eight neurons corresponding to eight sensors. There are 24 neurons in the hidden layer and just one neuron in the output layer.

Second, the developed electronic nose system was evaluated using SVM. Different kernel functions were tested and finally, radial basis kernel functions were used to project the training data to a space that maximized the margin hyperplane (*i.e.*, the separation between rotten beef and rotten fish samples). The value of the regularization parameter C for the SVM was set to 20.

Finally, the third classifier, KNN, was used to test the ability of the developed electronic nose setup to identify spoiled meat. For the KNN classifier defined in the previous section (see Equation (4)), we need to initialize the value of K. In this study, the value is 5.

### 4.3. Performance Metrics

The applied classifiers can be evaluated on the basis of three metrics, *i.e.*, accuracy, sensitivity, and specificity. All of the above-mentioned metrics can be computed on the basis of four factors, *i.e.*, true positive (tp), false positive (fp), true negative (tn), and false negative (fn). The definition of the above-mentioned evaluation factors are as follows:

True positive (tp): This factor means that ground truth is positive, and the predicted value is also positive. For example, the ground truth is beef and the predicted item is also beef.
False positive (fp): This factor means that ground truth is positive, and the predicted value is negative. For example, the ground truth is beef, but it is misidentified.
False negative (fn): Ground truth is negative, but the predicted value is positive. For example, the ground truth is a non-beef spoiled item, but it is incorrectly declared as beef.
True negative (tn): Ground truth is negative, and the predicted value is also negative. For example, the ground truth is a non-beef spoiled item, but it is correctly declared as non-beef.

The above-mentioned measures are summarized in terms of evaluation factors in Table 3.

### 4.4. Comparison

The classification of the meat samples, such as rotten beef and fish, was evaluated with ANN, SVM, and KNN in terms of accuracy, sensitivity, and specificity. Accuracy refers to the percentage of predictions that are correct. Sensitivity refers to the percentage of positive labeled instances that are predicted as positive, such as how many times a system identifies rotten beef when it is actually rotten beef. Specificity refers to the percentage of negatively labeled instances that are predicted as negative, such as when beef is not rotten and is also declared not rotten. Both metrics, sensitivity and specificity, collectively are the representatives of a classifier's reliability. The testing samples are divided into two groups. The first group consists of samples of fresh fish and rotten beef, whereas the second group consists of fresh beef and rotten fish samples. The goal of the system is to identify the type of rotten meat. Classification results using ANN, SVM, and KNN are given in Tables 4–6, respectively. Finally, the accuracy of all three applied techniques is listed in Table 7.

The results indicate that KNN is a more reliable technique than SVM or ANN because it has relatively higher values of sensitivity and specificity for both kinds of samples. Additionally, the accuracy rate is 85% for ANN, 94.5% for SVM, and 96.2% for KNN, showing that KNN has the highest performance. Although KNN is the simplest of all the evaluated techniques for the developed system, a survey of the results reveals that it is still the most suitable candidate to use as a pattern classification algorithm.

### 4.5. Discussion

The presented data suggest the possibility of using an electronic nose for food inspection, especially with meat. The experimental data is divided into two groups: (1) rotten beef with fresh fish, and (2) rotten fish with fresh beef. For group 1, out of 684 samples, the system correctly identified 670 (ANN), 639 (SVM), and 639 (KNN) samples as rotten beef and incorrectly identified 156 (ANN), 20 (SVM), and 0 (KNN) rotten beef samples as fresh beef. Consequently, ANN has the highest sensitivity of 97.2% but the lowest specificity of 69.59% whereas KNN has the highest specificity of 100% and a reasonable sensitivity of 93.42%. Likewise, in the experiment involving fish, KNN performed significantly better than ANN and SVM.

**Figure 5.**
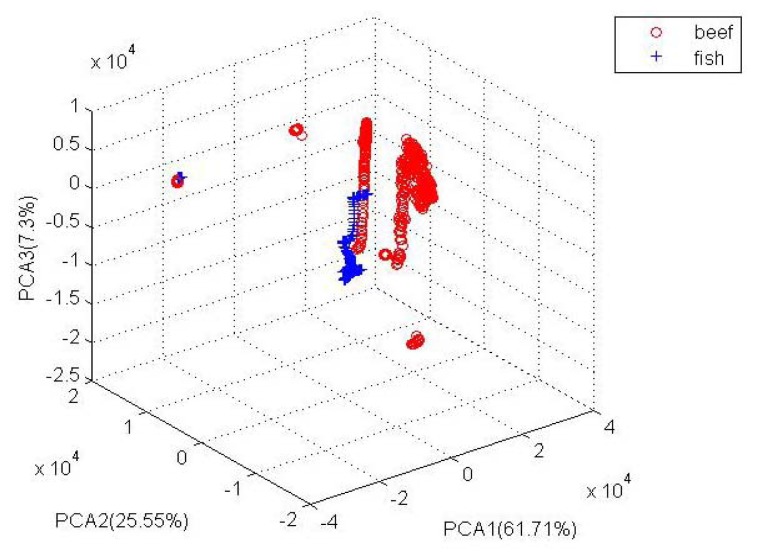
PCA plot of odor of both rotten beef with fresh fish and rotten fish with fresh beef.

Figure 5 shows a three-dimensional projection of the PCA results of all data points regarding the odor of the meat ample used for the experiments. The three dimensions explained almost 95% of the variation in the data, 61.61% for PCA1, and 25.55% for PCA2 and 7.3% for PCA3.

From our experimental results, following inferences can be made:

- The figure shows good recognition boundaries for the both kind of meat samples, and a high classification accuracy percentage was therefore expected.
- A number of data points from the both classes (*i.e.*, rotten beef with fresh fish and rotten fish with fresh beef) were mixed; therefore, a certain degree of misclassification was expected.
- The proposed portable E-Nose system (implemented with KNN) achieved an accuracy of 96.6% when identifying these two kinds of rotten meat.
- The odor patterns of different rotten meat were distinguishable, enabling the possibility of recognizing the malicious odor meat.

## 5. Conclusions

This article proposed a food inspection system that can be used to control the quality of food items in supermarkets. The system structure, working procedure, odor signal preprocessing, and pattern classification methods were introduced. To evaluate the proposed system's performance, fish and beef samples were taken and decayed for the sake of experiment. The results show that the proposed system is a very effective tool for assisting officers in food market inspections. Although the current pattern classification method produces satisfactory results, it should still be possible to further improve the classification accuracy and speed of operation by selecting more appropriate features in the preprocessing step. Future research should investigate how to select the most suitable features in the preprocessing step, and the scope of the experiment could also be extended to a larger number of food items.

## Figures and Tables

**Table 1.** Sensors with their measurable quantity.

**Sensors**	**Measurable Quantity**
MiCS-2610	O_3_
GSLS61	LPG/NG
GSNT11	NOx
MQ3	Alcohol
GSAP61	Smoke
GSBT11	VOC
GSET11	CO
TGS826	NH_3_

**Table 2.** Dataset for the experiment.

**Type of samples**	**Total samples**	**Training samples**	**Test samples**
Beef	784	100	684
Fish	588	75	513

**Table 3.** Definitions of evaluation terms.

		**Test Outcome**		**Accuracy**	**Sensitivity**	**Specificity**

		**Positive**	**Negative**			

**Actual Condition**	**Positive**	*tp*	*fp*	$\frac{tp+tn}{tp+fp+tn+fn}$	$\frac{tp}{tp+fn}$	$\frac{tn}{tn+fp}$
	**Negative**	*fn*	*tn*			

**Table 4.** Classification results using ANN.

		**Training samples**	**Test samples**	**Test outcome**		**Sensitivity**	**Specificity**

				**Positive**	**Negative**		

**Beef**	**Positive**	100	684	670	156	97.2%	69.59%
	**Negative**	75	513	14	357		

**Fish**	**Positive**	75	513	357	14	69.5%	97.2%
	**Negative**	100	684	156	670		

**Table 5.** Classification results using SVM.

		**Training samples**	**Test samples**	**Test outcome**		**Sensitivity**	**Specificity**

				**Positive**	**Negative**		

**Beef**	**Positive**	100	684	639	20	93.42%	96.1%
	**Negative**	75	513	45	493		

**Fish**	**Positive**	75	513	493	639	96.1%	93.42%
	**Negative**	100	684	20	45		

**Table 6.** Classification results using KNN.

		**Training samples**	**Test samples**	**Test outcome**		**Sensitivity**	**Specificity**

				**Positive**	**Negative**		

**Beef**	**Positive**	100	684	639	0	93.42%	100%
	**Negative**	75	513	45	513		

**Fish**	**Positive**	75	513	513	45	100%	93.42%
	**Negative**	100	684	0	639		

**Table 7.** Accuracy results of ANN, SVM, and KNN.

	**ANN**	**SVM**	**KNN**
**Beef**	85.7%	94.5%	96.2%
**Fish**	85.7%	94.5%	96.2%

## References

[1] Gram L., Ravn L.L., Rasch M., Bruhn J.B., Christensen A.B., Givskov M. (2002). Food spoilage-interactions between food spoilage bacteria. Int. J. Food Microbiol..

[2] Edwards D.S., Johnston A.M., Mead G.C. (1997). Meat inspection: An overview of present practices and future trends. Vet. J..

[3] Powers S.B. (2004). Downwind Air Quality Measurements from Poultry and Livestock Facilities.

[4] Pan L., Yang S.X. (2009). An electronic nose network system for online monitoring of livestock farm odors. IEEE/ASME Trans. Mech..

[5] Gardner J.W., Barlett P.N. (1994). A brief history of electronic noses. Sens. Actuators B Chem..

[6] Yogeswaran U., Chen S. (2008). A review on the electrochemical sensors and biosensors composed of nanowires as sensing material. Sensors.

[7] Wilson A.D., Baietto M. (2009). Advances in electronic nose technologies. Sensors.

[8] Ehret B., Safenreiter K., Lorenz F., Biermann J. (2011). A new feature extraction method for odour classification. Sens. Actuators B Chem..

[9] Byun J.S., Min J.S., Kim I.S., Kim J.W., Chung M.S., Lee M. (2003). Comparison of indicators of microbial quality of meat during aerobic cold storage. J. Food Protect..

[10] Borch E., Kant-Muermans M.L., Blixt Y. (1996). Bacterial spoilage of meat and cured meat products. Int. J. Food Microbiol..

[11] Papadopoulos O.S., Panagou E.Z., Mohareb F.R., Nychas G.-J.E. (2012). Sensory and microbiological quality assessment of beef fillets using a portable electronic nose in tandem with support vector machine analysis. Food Res. Int..

[12] Ghasemi-Varnamkhasti M., Mohtasebi S.S., Siadat M., Balasubramanian S. (2009). Meat quality assessment by electronic nose (machine olfaction technology). Sensors.

[13] Pearce T.C., Schffman S.S., Nagle H.T., Gardner J.W. (2003). Handbook of Machine Olfaction.

[14] Scientec Lab Center Co. Ltd. (2009). Development of the Odor Measurement and Monitoring System Based on Web GIS.

[15] Wilson A.D., Baietto M. (2011). Advances in electronic nose technologies developed for biomedical applications. Sensors.

[16] Barbri N.E., Llobet E., Bari N.E., Correig X., Bouchikhi B. (2008). Electronic nose based on metal oxide semiconductor sensors as an alternative technique for the spoilage classification of red meat. Sensors.

[17] Balasubramanian S., Panigrahi S., Longue C.M., Gu H., Marchello M. (2009). Neural networks-integrated metal oxide-based olfactory system for meat spoilage identification. J. Food Eng..

[18] Winquist F., Hornsten E.G., Sundgren H., Lundstrom I. (1993). Performance of an electronic nose for quality estimation of ground meat. Meas. Sci. Technol..

[19] Bothe D.D.H., Arnold J.W. (2002). Electronic nose analysis of volatile compounds from poultry meat samples, fresh and after refrigerated storage. J. Sci. Food. Agric..

[20] Green G., Chan A., Goubran R. Tracking Food Spoilage in the Smart Home Using Odour Monitoring.

[21] O'Connell M., Valdora G., Peltzer G., Negri R.M. (2001). A practical approach for fish freshness determinations using a portable electronic nose. Sens. Actuators B Chem..

[22] Mateo A., Soto F., Villarejo J.A., Dorda J.R., De la Gandara F., Garcia A. (2006). Quality analysis of tuna meat using an automated color inspection system. Aquacult. Eng..

[23] Gutierrez-Osuna R. (2002). Pattern analysis for machine olfaction: A review. IEEE Sensors.

[24] Eungyeong K., Seok L., Taikjin L., Beom J.S., Jungho L., Young T.B., Hyung S.K. An Intelligent Real-Time Odor Monitoring System Using a Pattern Extraction Algorithm.

[25] Aleksander I., Morton H. (1990). An Introduction to Neural Computing.

[26] Zhang G.P. (2000). Neural networks for classification: A survey. IEEE Trans. Syst. Man Cybernet..

[27] Lippmann R.P. (1987). Introduction to computing with neural nets. IEEE ASSP Mag..

[28] Gardner J.W., Hines E.L., Wilkinson M. (1990). The application of artificial neural networks in an electronic nose. Meas. Sci. Technol..

[29] Sundgren H., Winquist F., Lukkari I., Lundstrom I. (1993). Artificial neural networks and gas sensor arrays: Quantification of individual components in a gas mixture. Meas. Sci. Technol..

[30] Vapnik V.N. (1995). The Nature of Statistical Learning Theory.

[31] Hsu C.W., Lin C.J. (2002). A comparison of methods for multiclass support vector machines. IEEE Trans. Neural Netw..

[32] Cho N., Kim E-Y. (2011). Enhanced voice activity detection using acoustic event detection and classification. IEEE Trans. Consum. Electron..

[33] Dasarathy B.V. (1991). Nearest Neighbor (NN) Norms: NN Pattern Classification Techniques.

